# Solvent dispersibility of two-dimensional particles with pseudo- and permanently interlocked polyethylene oxide brushes

**DOI:** 10.1039/d6ra00421k

**Published:** 2026-05-18

**Authors:** Shuntaro Uenuma, Di Liu, Kohzo Ito

**Affiliations:** a International Center for Young Scientists, National Institute for Materials Science 1-2-1, Sengen Tsukuba Ibaraki 305-0047 Japan; b Department of Advanced Materials Science, Graduate School of Frontier Sciences, The University of Tokyo Kashiwa City Chiba 277-8561 Japan; c Research Center for Macromolecules and Biomaterials, National Institute for Materials Science 1-2-1 Sengen Tsukuba Ibaraki 305-0047 Japan

## Abstract

The solvent dispersibility of two-dimensional particles with pseudo- and permanently interlocked polyethylene oxide brushes was investigated. Their dispersibility was determined by desorption or retention of the polyethylene oxide axis of two-dimensional particles. This study provides new insight into controlling the dispersion and aggregation of particles.

## Abstract 2

The solvent dispersibility of two-dimensional particles with pseudo- and permanently interlocked polyethylene oxide brushes was investigated.

## Graphical abstract

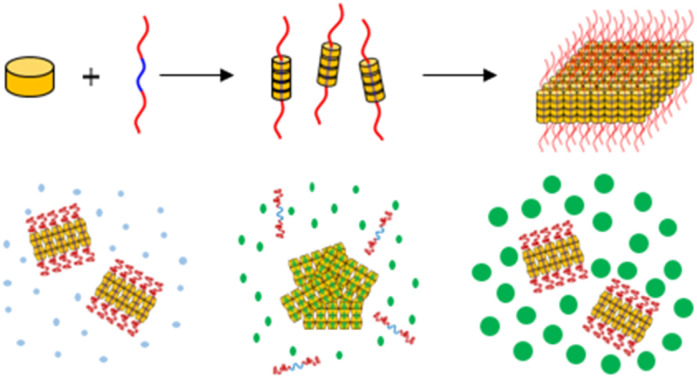

The dispersibility of particles with sizes ranging from several nanometers to micrometers in solvents is important for practical applications in the food industry, pharmaceuticals, nanocarriers, cosmetics, inks, and glues.^1–5^ van der Waals interactions generally occur between particles in this size range; therefore, steric repulsions of grafted polymer chains are widely used for ensuring high colloidal stability.^6–8^ Polymer chains covalently grafted to particles endow them with long-term stability. Meanwhile, controlling the dispersion and aggregation of particles is also important for the development of stimuli-responsive materials for environmental remediation, biological applications, sensing, and photonics.^9–11^ For example, proteins containing charged and hydrophobic groups enable charge control in response to pH, thereby regulating their dispersion and aggregation.^12–14^ Poly(*N*-isopropylacrylamide) possesses a low critical solution temperature of 32 °C that allows a reversible transformation between the hydrophobic aggregated and hydrophilic solvated states.^15,16^ Photoisomerization-induced steric changes in azobenzene derivatives that occur on the particle surface can also be used to control the dispersion and aggregation states.^17–19^

Molecular architecture designs can also be used for controlling material properties. Mechanically interlocked structures, such as rotaxane-type interlocked molecules, possess unique functions, which include rotating, shuttling, and location switching, owing to the mobility of the ring and/or axis components.^20–22^ The design of interlocked structures does not compete with the introduction of functional chemical structures, allowing their simultaneous incorporation into a material. Therefore, the introduction and design of interlocked molecular structures on the particle surface may be effective for controlling dispersion and aggregation.

Cyclodextrin-based (pseudo-)polyrotaxane self-assembly systems are promising for the development of stimuli-responsive nano- and micro-scale materials.^23–27^ Our group has reported the fabrication of nanosheet particles with a rotaxane structure, *i.e.*, pseudo-polyrotaxane nanosheets (PPRNS) (Fig. 1a).^28^ They are prepared by mixing β-cyclodextrin (β-CyD) and poly(ethylene oxide)_75_-*b*-poly(propylene oxide)_29_-*b*-poly(ethylene oxide)_75_ (EO_75_PO_29_EO_75_) in water. β-CyD selectively covers the central PO region and this inclusion complex assembly in a nanosheet particle consisting of a single-crystal layer of β-CyD with a thickness of 11 nm (equal to the length of a PO segment) and interlocked EO brushes on its surface.^29,30^

**Fig. 1:**
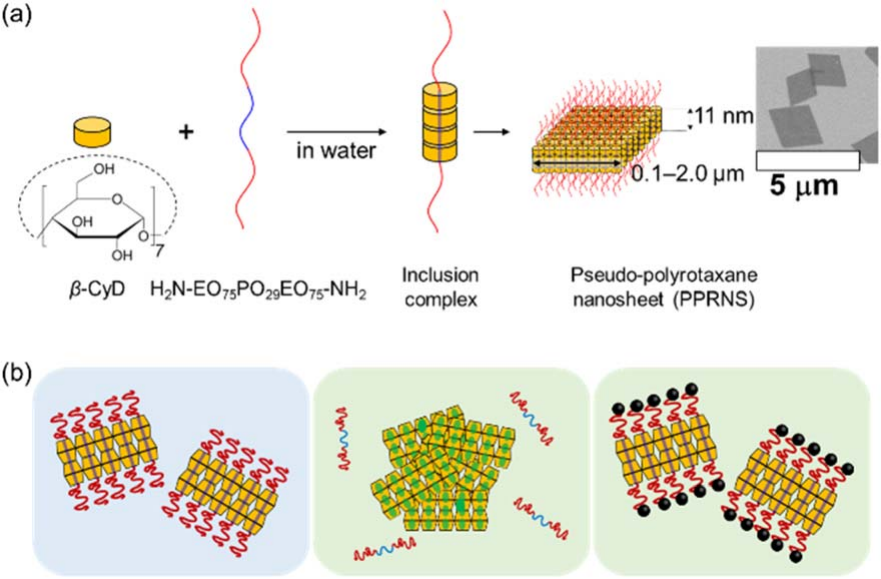
(a) Schematic of PPRNS formation. (b) Schematics of the dispersion behavior of PPRNS and capped PPRNS in solvents. The dispersion and aggregation of PPRNS are governed by the retention (left) or desorption (center) of EO_75_PO_29_EO_75_ from the β-CyD crystalline core, while capped PPRNS retain EO_75_PO_29_EO_75_ in various solvents, resulting in stable dispersion (right).

PPRNS represent an analyzable model system with a well-defined morphology and molecular structure. In this study, we investigated the dispersion and aggregation behavior of PPRNS and axis-end-capped PPRNS (capped PPRNS) containing pseudo- and permanently interlocked EO brushes in various solvents, focusing on the behavior of the axis polymer (Fig. 1b). Amine-terminated EO_75_PO_29_EO_75_ was used for the preparation of PPRNS, enabling subsequent capping *via* a click reaction between the NH_2_ groups and bulky trimethylolpropane triglycidyl ether in water.^30^ The results revealed that the dispersion and aggregation of PPRNS are governed by the retention and desorption of EO_75_PO_29_EO_75_ from the CyD crystalline core and that the capped PPRNS can hold the EO_75_PO_29_EO_75_ in various solvents, resulting in good dispersion.

The solvent for PPRNS and capped PPRNS was varied from water to organic solvents following the procedure described in Fig. 2a, and their dispersibility was evaluated *via* optical microscopy (OM, SI S3). The behaviors of PPRNS and capped PPRNS in various solvents are summarized in Table 1. The PPRNS aggregated in many types of organic solvents, while the capped PPRNS exhibited good dispersion in almost all solvents, with the exception of hexane. The speeds of formation of aggregation were typically very fast (immediately after the addition, within several seconds).

**Fig. 2:**
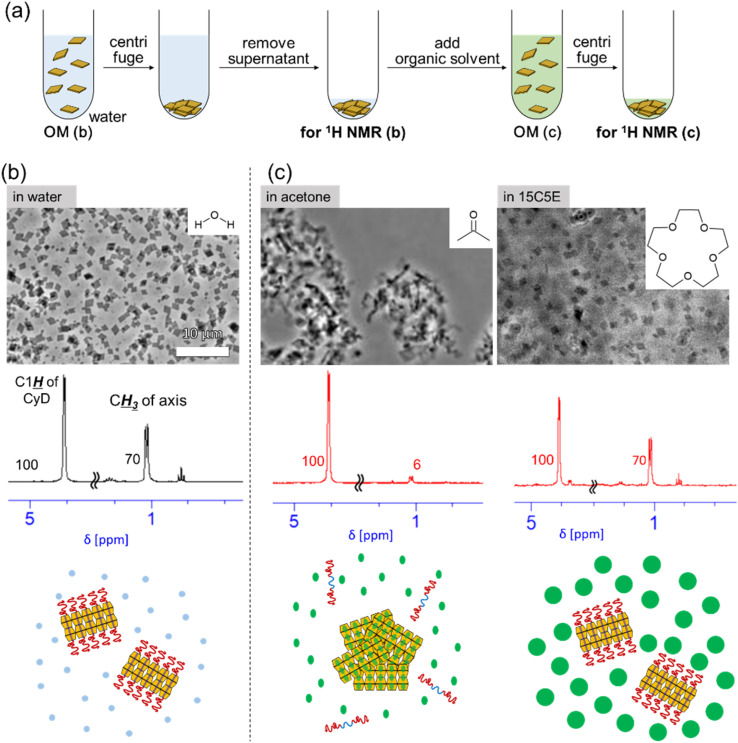
(a) Solvent exchange protocol for transferring the PPRNS dispersion from water to various organic solvents. OM images, ^1^H NMR spectra of particles collected *via* centrifugation (dissolved in DMSO-*d*_6_), and schematics of the dispersion or aggregation behavior of PPRNS in (b) water and (c) organic solvents (acetone and 15C5E) are shown. The numbers in the ^1^H NMR spectra represent integral values. The scale bar applies to all images.

**Table 1:** Dispersibility of PPRNS and capped PPRNS in a series of solvents^a^

	H_2_O	15C5E^f^	MeOH	EtOH	Acetone	THF^g^	DEGDME^h^	CHCl_3_	EtOAc^i^	PGMEA^j^	Hexane	DMSO
PPRNS	○	○	×^b^	×^b^	×^c^	×^c^	×^c^	×^d^	×^d^	×^d^	×^d^	Dissolved
Capped PPRNS	○	○	○	○	○	○	○	○^e^	○^e^	○^e^	×^e^	Dissolved

a ○: Dispersed. ×: not dispersed.

b Gradual morphological change to large crystal.

c Axes were desorbed.

d Low affinity of solvents for water is one reason for aggregation.

e Solvents were changed from MeOH (miscible for both organic solvents and water).

f 15-Crown-5-ether.

g Tetrahydrofuran.

h Diethylene glycol dimethyl ether.

i Ethyl acetate.

j Propylene glycol 1-monomethyl ether 2-acetate.

As representative examples, PPRNS aggregation in acetone and its dispersion in water and 15C5E are shown in Fig. 2b and c. The precipitated PPRNS were collected *via* centrifugation, and its composition was analyzed using ^1^H nuclear magnetic resonance (NMR). For PPRNS in water, the integral of the β-CyD peak was set to 100 as the standard, and that of the axis CH_3_ signal was 70. Meanwhile, for PPRNS in acetone, the integral of the axis was significantly reduced (6, Fig. 2c). This indicates the desorption of EO_75_PO_29_EO_75_ from the β-CyD cavity, which leads to particle aggregation. The desorption is caused by the high affinity of the axis for acetone. The EO_75_PO_29_EO_75_ in β-CyD cavity is thought to be replaced with solvent molecules. After being dispersed in acetone, PPRNS was dissolved in water, which also support that the fact of desorption of EO_75_PO_29_EO_75_. Meanwhile, PPRNS in 15-crown-5 ether (15C5E) (bulky liquid) was well dispersed (Fig. 2c) over one week. The ^1^H NMR results indicated that the axis polymer was retained within the PPRNS structure (Fig. 2c). This likely occurs because 15C5E has high affinity for the EO segment but cannot enter the β-CyD cavity owing to steric hindrance. As a result, the axial structure in PPRNS is maintained, leading to high dispersibility of PPRNS.

As a prerequisite for the dispersibility of PPRNS, the EO segments must be solvated; however, this can lead to desorption of EO_75_PO_29_EO_75_ from the β-CyD cavity, as observed in PPRNS dispersed in acetone. Because many types of organic solvent molecules are smaller than the β-CyD cavity, they can readily enter the cavity unless this process is highly unfavorable, leading to the desorption of the axis polymer and PPRNS aggregation. In contrast, 15C5E and water are unique solvents that keep PPRNS well dispersed. Although both solvents can solvate EO brushes, their penetration into the β-CyD cavity is energetically unfavorable, owing to steric hindrance for 15C5E and strong hydrogen bonding among water molecules for water.^31,32^

Next, the capped PPRNS was prepared by end-capping *via* a click reaction between NH_2_ groups of PPRNS and bulky trimethylolpropane triglycidyl ether in neutral water at room temperature.^30^ Its dispersibility was investigated (solvent exchange to a water-immiscible solvent was performed using the capped PPRNS dispersion in MeOH, which is miscible with both water and the organic solvent). The capped PPRNS exhibited good dispersibility in a range of organic solvents (OM images of capped PPRNS in acetone are shown in Fig. 3a, while those in other organic solvents are presented in SI S3). For capped PPRNS in acetone, compositional analysis *via*^1^H NMR indicated the retention of EO_75_PO_29_EO_75_ (Fig. 3b). These results suggest that the permanent interlocking of EO_75_PO_29_EO_75_ endows PPRNS with high dispersibility in various solvents by retaining the axis (Fig. 3c). Consistent with this interpretation, the capped PPRNS did not disperse in hexane (SI S3), where the PEO brushes are not solvated.

**Fig. 3:**
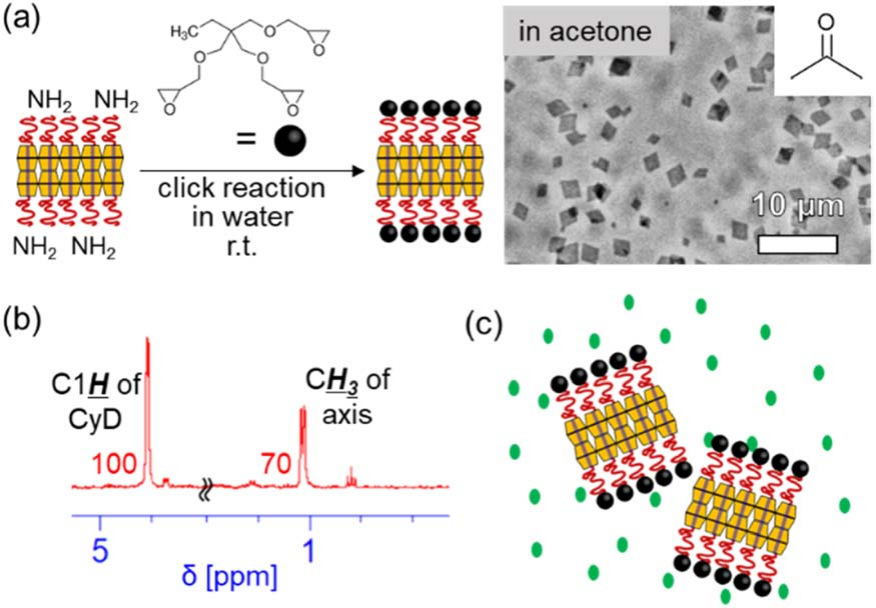
(a) Schematic of the synthesis of capped PPRNS from PPRNS and OM images of capped PPRNS dispersed in acetone. (b) ^1^H NMR spectrum of capped PPRNS in DMSO-*d*_6_ (the numbers denote the integrals). (c) Schematic of the PPRNS dispersion in acetone.

The morphology of PPRNS in ethanol (EtOH) and methanol (MeOH) was slowly changed from nanosheet to microcrystal (SI S2). This slow transformation (from 5 to 60 min) is probably caused by the balance among the low solubility of β-CyD, the low binding constant of the β-CyD cavity with MeOH and EtOH, and the slow axis desorption. Accurate prediction of PPRNS behavior requires additional quantitative studies. In contrast to PPRNS, the capped PPRNS particles remained stable for over one week.

Changing the solvent of PPRNS from water to CHCl_3_, EtOAc, PGMEA, or hexane induced aggregation. The immiscibility of these solvents with water makes the solvent exchange imperfect, and this is probably one reason for the aggregation. Despite being immiscible, for PPRNS in CHCl_3_, compositional analysis (^1^H NMR) indicated the desorption of the axis of PPRNS (the integral ratio of the axis CH_3_ signal to the β-CyD signal was reduced from 70/100 to 21/100 after solvent exchange). This implies that PPRNS aggregate in water-immiscible organic solvents that have high affinity for the axis polymer, even if the solvent exchange proceeds successfully.

The dispersion and aggregation behavior of PPRNS and capped PPRNS in various organic solvents have been systematically investigated. Based on these findings, the factors that determine the dispersion, aggregation, and structural behavior of PPRNS are outlined below.

(1) Affinity of solvent for PEO brushes: the solvent should have high affinity with the PEO moieties for dispersion. In hexane, capped PPRNS aggregate because of the poor solvation of the PEO brush.

(2) Retention and desorption of the axis polymer: for PPRNS in solvents that are good for PEO brushes, retention of the axis polymer within the cavity of the β-CyD crystal is crucial for maintaining dispersion. In contrast, PPRNS aggregate when the axis polymer is desorbed.

(3) Balance between axis-polymer retention and desorption: the stability of the axis polymer in the cavity of the β-CyD crystal is determined by the balance between the dissolution of the polymer from the cavity and the subsequent occupation of the cavity by solvent molecules. In most solvents, PPRNS aggregate as a result of axis-polymer dissolution with subsequent occupation by solvent molecules. In contrast, PPRNS retained the axis polymer and remained well dispersed in water and 15C5E, likely because these solvents do not penetrate the β-CyD cavity.

(4) End-capping: end-capping of the axis in PPRNS prevents desorption of the axis polymer from the cavity of the β-CyD crystal, resulting in stable dispersion in a wide range of solvents.

(5) Dissolution in DMSO: both PPRNS and capped PPRNS dissolve in DMSO owing to the high solubility of β-CyD. This enables compositional analysis of PPRNS and capped PPRNS *via* solution ^1^H NMR.

(6) Morphological transformation in ethanol or methanol: in ethanol or methanol, both the axis polymer and β-CyD of PPRNS may slowly dissolve, causing morphological transformation into large crystals. Further dynamic and kinetic analysis would be required to clarify these processes.

In this study, we examined the dispersion behavior of PPRNS and capped PPRNS, which are two-dimensional particles featuring pseudo- and permanently interlocked PEO brushes. The well-defined morphology and molecular structure of PPRNS enabled analysis of their dispersion and aggregation behavior, providing a foundation for interpretating, predicting, and controlling the dispersibility of particles with interlocked (including entangled) polymers. This study provides a new insight into controlling the dispersion and aggregation of particles.

## Conflicts of interest

There are no conflicts to declare.

## Supplementary Material

RA-016-D6RA00421K-s001

## Data Availability

The data supporting the findings of this study are available within the article or its supplementary information (SI). Supplementary information is available. See DOI: https://doi.org/10.1039/d6ra00421k.
