# Supplementary material for: Solvent dispersibility of two-dimensional particles with pseudo- and permanently interlocked polyethylene oxide brushes
Source: RSC Adv. 2026 May 18;16(29):26299–302. doi: 10.1039/d6ra00421k (PMC13185685; doi:10.1039/d6ra00421k)
Supplement: RA-016-D6RA00421K-s001 [file RA-016-D6RA00421K-s001.pdf]

Supplementary Information (SI) for RSC Advances.
This journal is © The Royal Society of Chemistry 2026

Supplementary Information

Solvent dispersibility of two-dimensional particles with pseudo- and permanently interlocked polyethylene oxide brushes

Shuntaro Uenuma[a,*], Di Liu[b], Kohzo Ito[b,c,*]

[a] International Center for Young Scientists, National Institute for Materials Science, Ibaraki 305-0047, Japan

[b] Department of Advanced Materials Science, Graduate School of Frontier Sciences, The University of Tokyo, Kashiwa City, Chiba 277-8561, Japan

[c] Research Center for Macromolecules and Biomaterials, National Institute for Materials Science (NIMS), 1-2-1 Sengen, Tsukuba, Ibaraki 305-0047, Japan

[S1] *General information* S2
[S2] *Sample preparation* S2
[S3] *Dispersibility of PPRNS in water and organic solvents* S3
[S4] *Dispersibility of capped PPRNS in water and organic solvents* S4

[S1] *General information*

β-cyclodextrin (CyD), dimethyl sulfoxide (DMSO)-d_6, methanol (MeOH), ethanol (EtOH), acetone, tetrahydrofuran (THF), ethyl acetate (EtOAc), and hexane were obtained from Fujifilm Wako Pure Chemical Corporation. Hydroxyl-terminated poly(ethylene oxide)$_{75}$-*b*-poly(propylene oxide)$_{29}$-*b*-poly(ethylene oxide)$_{75}$ ($EO_{75}PO_{29}EO_{75}$) triblock copolymer (Pluronic F68) was acquired from Sigma–Aldrich. Diethylene glycol dimethyl ether (DEGDME), propylene glycol 1-monomethyl ether 2-acetate (PGMEA), Tokyo Chemical Industry Co. 15-crown-5-ether was obtained from Kanto Chemical. All reagents were used as received.

Scanning electron microscopy (SEM) was conducted using a JEOL JSM-7800F instrument. ^{1}H nuclear magnetic resonance (NMR) spectra were collected using a JEOL JNM-AL400 instrument. Optical microscopy (OM) was performed using a Nikon ECLIPSE Ts2R instrument equipped with a Nikon DS-Fi3 camera.

[S2] *Sample preparation*

Amine-terminated $EO_{75}PO_{29}EO_{75}$

Amine-terminated $EO_{75}PO_{29}EO_{75}$ was synthesized following a previously reported method[1] and utilized to prepare PPRNS.

PPRNS

The PPRNS synthesis procedure followed a previously reported method with minor modifications. First, β-CyD (18 mg/mL) was dissolved in water. Then, 8.0 mg of the amine-terminated $EO_{76}PO_{29}EO_{76}$–NH_2 was added to the solution, which was subsequently stirred for over a month.

Capped PPRNS

The capped PPRNS preparation procedure followed a previously reported method with minor modifications[2]. Trimethylolpropane triglycidyl ether (20 equivalents vs. axis polymer [mol/mol]) was added to a PPRNS water dispersion and reacted for 1 week at 25 °C. Next, 1000 μL of the crude reaction mixturewas centrifuged, and 950 uL of the supernatant was replaced with ultrapure water. This purification step was repeated three times.

Solvent exchange of PPRNS and capped PPRNS for OM and ^{1}H NMR analysis

To conduct a dispersity test, 200 μL of the water dispersion of PPRNS or capped PPRNS was centrifuged, and 180 μL of supernatant was removed. Subsequently, 480μL of an organic solvent was added. The speeds of formation of aggregation were typically very fast (immediately after the addition, within several seconds). In the cases of EtOAc, $CHCl_3$, PGMEA, and hexane, replacement of water did not occur, because of the extremely low miscibility. For capped PPRNS, the solvent (water) was replaced with MeOH, which was subsequently replaced with water-immiscible solvent (MeOH is miscible for these solvent and water).

To determine the compositions of PPRNS and capped PPRNS, 200 μL of the water dispersion of PPRNS or capped PPRNS was centrifuged, 150 μL of the supernatant was removed, and 850 μL of an organic solvent was added, followed by storage for 1 d. These samples were centrifuged, and precipitations were collected and dried. The obtained white powder was dissolved in DMSO-d_6 at a concentration of 1 mg/mL.

[S3] *Dispersibility of PPRNS in water and organic solvents*

The dispersibility of PPRNS in water and organic solvents was evaluated via OM. Images of PPRNS in various solvents are shown in Fig. S1.

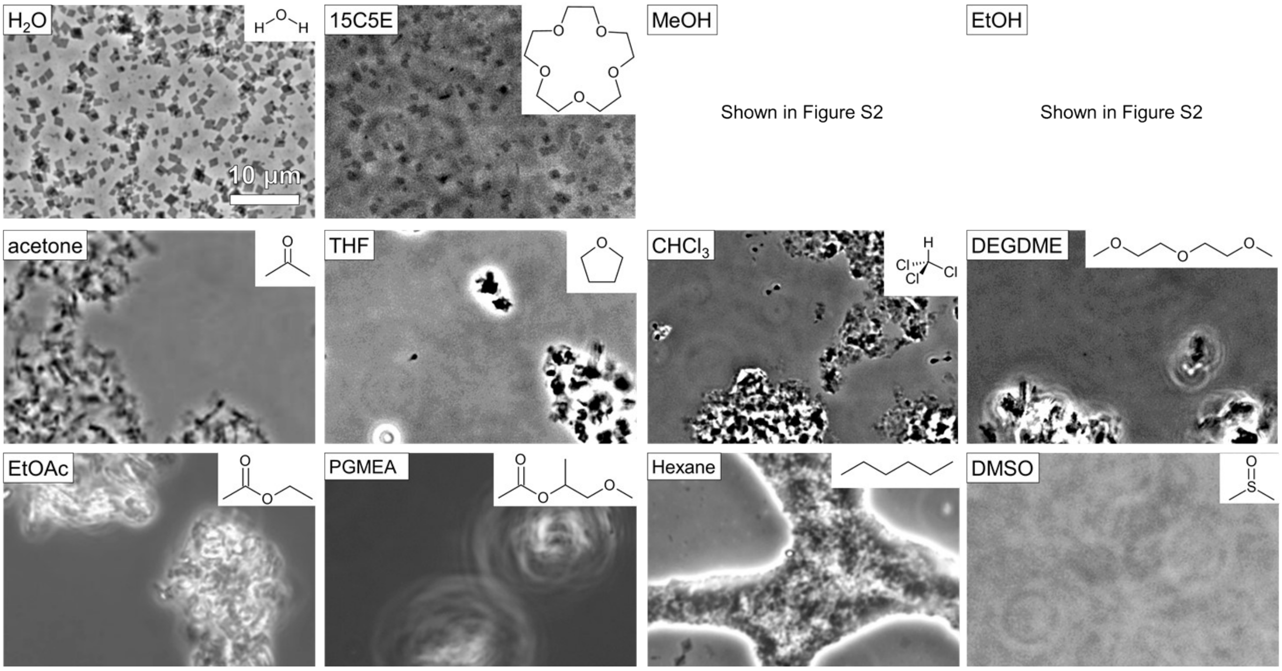

Fig. S1. OM images of PPRNS in water and organic solvents. The scale bar applies to all images.

PPRNS were dispersed and slightly dissolved (as indicated by the disordered shape of particles) in ethanol (a) and methanol (b) within a relatively short time (5 min) (Fig. S2). Then, after a long time (>60 min), the nanosheet morphology was changed from two dimensional particles to microcrystals with dimensions of >10 μm. This implies the solvent-mediated transformation of crystals based on the slight solubility of β-CyD.

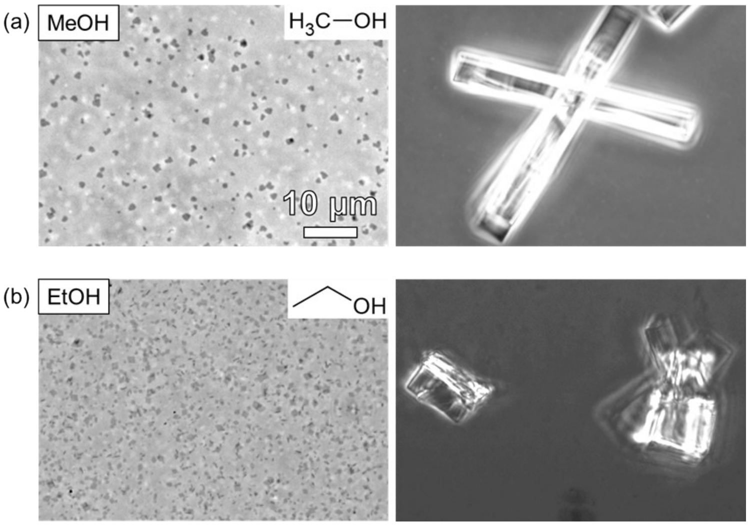

Fig. S2. OM images of PPRNS in (a) ethanol and (methanol) obtained after 5 min (left) and 60 min (right). The scale bar applies to all images.

[S4] *Dispersibility of capped PPRNS in water and organic solvents*

The dispersibility of capped PPRNS in water and organic solvents was evaluated via OM. Images of capped PPRNS in various solvents are shown in Fig. S3.

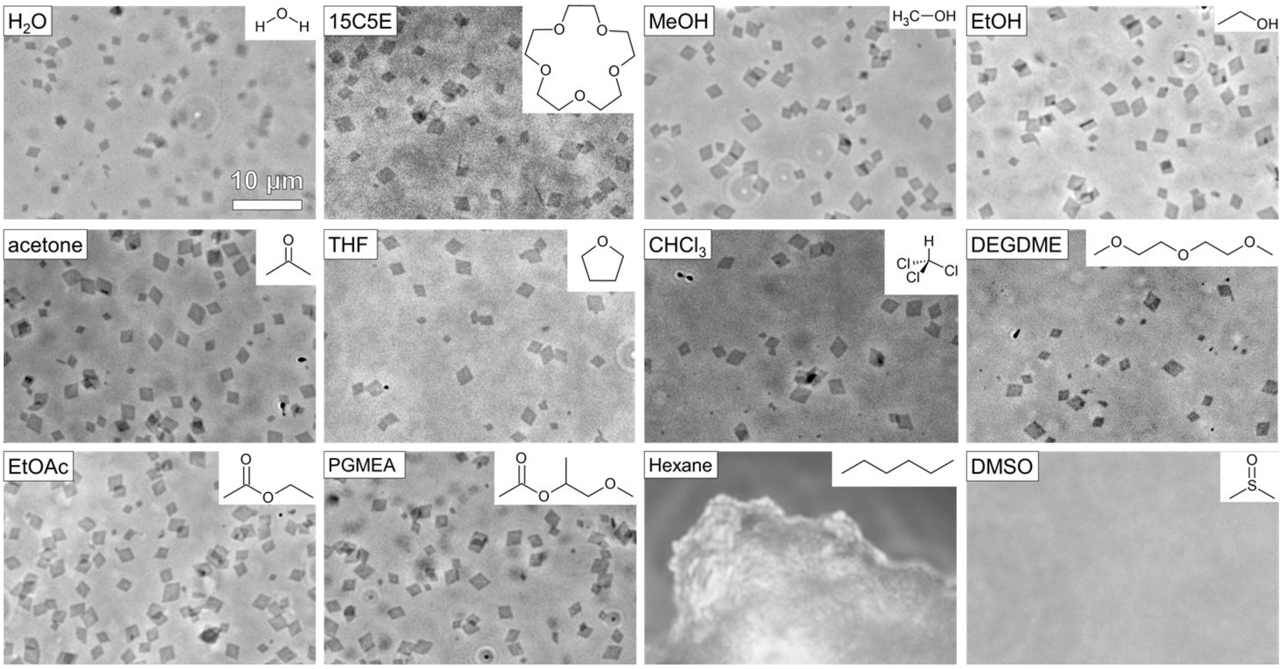

Fig. S3. OM images of capped PPRNS in water and organic solvents. The scale bar applies to all images.

[1] S. Uenuma, R. Maeda, H. Yokoyama, K. Ito, *ACS Macro Lett.*, 2021, **10**, 237.

[2] S. Uenuma, D. Liu, C. Liu, S. Ando, H. Yokoyama, K. Ito, *ACS Sustainable Chem. Eng.*, 2024, **12**, 18600
